# Effect of Graphene Oxide Modified with Organic Amine on the Aging Resistance, Rolling Loss and Wet-Skid Resistance of Solution Polymerized Styrene-Butadiene Rubber

**DOI:** 10.3390/ma13051025

**Published:** 2020-02-25

**Authors:** Songhan Wan, Xiaobin Lu, Hongguo Zhao, Songbo Chen, Shuwei Cai, Xianru He, Rui Zhang

**Affiliations:** 1 School of Materials Science and Engineering, Southwest Petroleum University, Chengdu 610500, China; songhanwan@foxmail.com (S.W.); xblu1009@163.com (X.L.); chen756424838@foxmail.com (S.C.); victor8951@foxmail.com (S.C.); 2 Petrochemical Research Institute, PetroChina, Lanzhou 730060, China; zhaohongguo@petrochina.com.cn; 3 Institute für Physik, Universität Rostock, 18051 Rostock, Germany; rui.zhang@uni-rostock.de

**Keywords:** p-phenylenediamine (PPD), graphene oxide (GO), solution polymerized styrene-butadiene rubber (SSBR), aging resistance, rolling loss, wet-skid resistance

## Abstract

Graphene oxide (GO) was modified by p-phenylenediamine (PPD), aiming at improving the wet-skid resistance and reduce the rolling loss of solution polymerized styrene-butadiene rubber (SSBR). PPD with amino groups enabled GO to obtain anti-aging function. The structure of modified GO (PPD-GO) was characterized by Fourier transform infrared spectroscopy (FTIR), X-ray diffraction (XRD) and Raman spectroscopy. Mechanical tests showed that the mechanical properties of SSBR before and after aging were improved by adding PPD-GO. The results of thermogravimetric-differential scanning calorimeter synchronization analysis (TGA-DSC) indicated that SSBR/PPD-GO obtained good thermo-oxidative stability. The dynamic mechanical analysis (DMA) of SSBR composites showed that the mechanical loss factor (tanδ) peak moved to high temperature with the content of PPD-GO. The tanδ values of SSBR composites showed that it had a good effect on improving the wet-skid resistance and reducing the rolling loss of SSBR by adjusting the content of PPD-GO. In particular, with the addition of 4 phr GO, SSBR was effectively improved in mechanical properties, aging resistance, wet-skid resistance and low rolling loss.

## 1. Introduction

Solution polymerized styrene-butadiene rubber (SSBR), with good elasticity, abrasion resistance, low rolling loss and wet-skid resistance, is widely used in the field of tread adhesive [1,2]. With the continuous development of the tire industry, higher requirements are put forward for the comprehensive performance of tread adhesive. The unsaturated double bonds make it easy for the aging resistance of SSBR to be decreased in the thermal-oxidative environment [3,4]. Therefore, it is necessary to improve the aging resistance while reducing the rolling loss and improving the wet-skid resistance of SSBR.

In the past decades, nanoparticles have received increasing interest owing to its unique structural and surface properties. There are many nanoparticles have been applied to improve the performances of rubber, such as graphene [5,6,7,8], layered double hydroxide [9,10], nano silica [11], carbon nano tubes [12], montmorillonite [13,14], ect. The properties of nanocomposites are better than that of conventional composites in the same composition due to the quantum effect and surface effect caused by the small size and large specific surface area. Graphene is a kind of two-dimensional carbon nanoparticle with high mechanical strength, excellent thermal and electrical conductivities [15,16,17]. According to researches, graphene allows rubber to achieve better overall performances. Yingyan Mao [18] prepared GO/silica/styrene-butadiene rubber composites. The wet-skid resistance of the composites increased, and the rolling loss decreased by about 10% when GO was added with a volume fraction of 0.6%. Chengpeng Li [19] modified natural rubber with graphene and found that the tensile strength of natural rubber increased by 9% when the addition of graphene accounted for only 0.07% of the volume fraction. S.H. Song [20] found that the thermal conductivity of styrene-butadiene rubber composites increased by about 20% when the addition amount of graphene was only 5% of the mass fraction, which far exceeding that of carbon black and other fillers of the same amount.

Graphene is easy to agglomerate in the polymer matrix due to the large specific surface and strong interaction between layers [21]. Therefore, many researches paid attention to modify graphene to improve its dispersibility in the polymer matrix. Graphene oxide (GO) is the derivative of graphene, with plenty of oxygen-containing groups, such as hydroxyl, epoxy group and carboxyl groups, which offers potential for the preparation of modified graphene [16,17]. Therefore, chemical grafting is an effective method to obtain modified GO [22,23,24,25,26]. For example, it was reported that alkylamine was covalently grafted to GO. The dispersion of GO in the rubber matrix was significantly improved [27]. 

Diamine grafted onto GO is an effective way to improve the thermostability and anti-aging property of polymers [6,28,29]. Junjun Zhou et al. found that the thermal oxidation resistance of SBR was effectively promoted by graphene oxide (GO) functionalized with the 4-aminodiphenylamine [30]. Zhong used an efficient one-step approach, which was developed to simultaneously reduce and functionalize GO via N-1, 3-dimethylbutyl-N′-phenyl-p-phenylenediamine. It was found that the SBR adding with the functionalized graphene exhibited high long-term thermo-oxidative aging resistance [31]. P-phenylenediamine (PPD), with the anti-aging amino groups, is not only beneficial to improve the interaction between GO and SSBR matrix, but also can endow GO with anti-aging function [32,33]. Many researches devoted to enhancement of polymers properties by modified graphene oxide, but most of them only looked at one aspect of performance improvement, such as mechanical properties, thermal stability, and so on. 

In the present study, GO was grafted by PPD to obtain the functionalized particle, PPD-GO. PPD-GO was added into SSBR, aiming at improving the wet-skid resistance and reducing rolling loss while improving the aging resistance of SSBR.

## 2. Materials and Methods 

### 2.1. Materials

The SSBR with styrene content 25.7% was obtained from China National Petroleum Corporation (CNPC) Lanzhou Petrochemical Company (Lanzhou, China). Graphite oxide was supplied by the Sixth Element Materials Technology co, LTD. (Changzhou, China). Zinc oxide (ZnO), sulfur, stearic acid, NH_3_·H_2_O and absolute ethyl alcohol were supplied by Chengdu Kelong Chemical Reagent co. LTD (Chengdu, China). P-phenylenediamine (PPD) was purchased from China Aladdin Industrial Corporation (Shanghai, China). Carbon black was obtained from Hongying carbon black factory (Chengdu, China). N-tert-butyl-2-benzothiazole sulfonamide (TBBS) was purchased from Duba new material technology co. LTD (Dongguan, China). 

### 2.2. The Synthesis of PPD-GO

**Figure 1:**
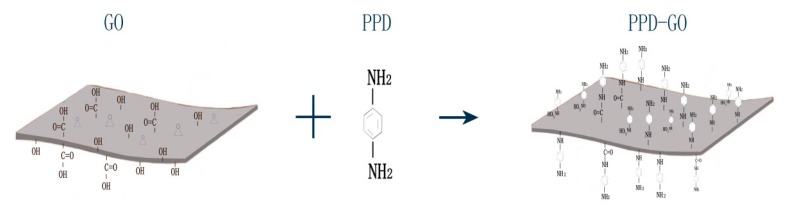
The Schematic diagram of the synthesis of p-phenylenediamine–graphene oxide (PPD-GO).

GO was obtained by mechanical ultrasonic stripping of graphite oxide. 1 g of graphite oxide was added into 1000 mL deionized water for ultrasonic dispersion (Huashen science and technology equipment co. LTD; Shen Zhen, China), with time of 2 h and the ultrasonic frequency was 3.5 kHz. The ultrasound products were freeze-dried to obtain the GO. The pH of 1000 mL deionized water was adjusted to about 10 by dropping NH_3_·H_2_O. Then 1 g of GO and 2 g of PPD were added for reaction at a temperature of 80 °C and a time of 12 h. Finally, the reaction products were filtered, rinsed and freeze-dried to obtain the PPD-GO particles. The Schematic diagram of the synthesis of PPD-GO was showed in Figure 1.

### 2.3. The Preparation of SSBR Composite

SSBR, particles and additives were mixed in a two-roll mixing mill (Guangdong Zhanjiang Rubber and Plastic Machinery Factory, Zhanjiang, China). The compositions of the SSBR composites were shown in the Table 1. The vulcanizing of the mixed rubber was carried out with the flat vulcanizer (Shanghai Rubber Machinery Factory, Shanghai, China) under a pressure 10 MPa at 145 °C for 35 min.

### 2.4. Characterization

Fourier transform infrared spectra (FTIR) analysis was carried out by the Thermo Fisher Scientific Nicolet iS50 infrared spectrometer (Waltham, MA, USA) with the scanning speed of 4 cm^−1^/min from 500 cm^−1^ to 4000 cm^−1^. KBr pellets were used in the test.

X-ray diffraction (XRD) diffractograms were obtained using a PANalytical B.V. X Pert PRO MPD X-ray diffractometer (PANalytical, Almelo, The Netherlands), equipped with Cu Kα radiation (λ = 0.154 nm). The scanning diffraction angle was from 3° to 70° with a scanning rate of 4°/min. 

Raman analysis was characterized by the spectrometer of ID Raman micro IM-52AV300 (Weihai Optical Instrument). The excitation source was He-Ne laser with a wavelength of 785 nm and a scanning range of 200 ~ 2000 cm^−1^.

Scanning electron microscopy (SEM) analysis was performed on a JSM-6360LV instrument (Japan Electronics Co, Tokyo, Japan) to study the fracture surface morphology of SSBR composites. 

Tensile test was conducted on a MTS CMT 6104 universal tensile testing machine (MTS Systems (China) Co., Ltd., Shanghai, China) according to the standard of GB/ t528-2009. The samples were cut into a dumbbell shape with thickness of 2 mm, width of 6 mm and the middle standard length of 25 mm.

Thermogravimetric-differential scanning calorimeter synchronization analysis (TGA-DSC) was carried out on a NETZSCH STA449F3 Jupiter instrument (NETZSCH, Bavaria, Germany) with the heating rate of 10 °C /min from 40 °C to 800 °C. The measurements were done under oxygen atmosphere with a gas flow rate of 20 mL/min.

Dynamic mechanical analysis (DMA) was carried out on a TA Q800 apparatus (TA Instruments, New Castle, DE, USA) with heating rate of 3 °C/min from −80 °C to 60 °C. The amplitude was 15 μm, and the frequency was 1 Hz. The size of the sample was 60 mm long, 12.8 mm wide and 2 mm thick. 

## 3. Results and Discussion

### 3.1. The Characterization of Structure of PPD-GO

**Figure 2:**
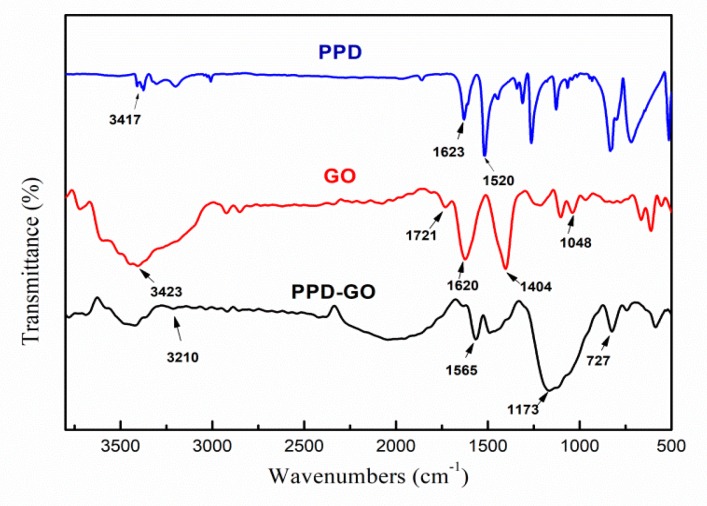
Fourier transform infrared (FTIR) spectra of PPD, GO and PPD-GO.

The PPD-GO was confirmed by FTIR spectroscopy. As shown in Figure 2 and Table 2, the curve of GO showed two peaks at 3423 cm^−1^ and 1404 cm^−1^, which corresponding to the hydroxyl and carboxyl, respectively. The peaks at 1721 cm^−1^, 1620 cm^−1^ and 1048 cm^−1^ were attributed to C=O in carboxyl group, C=C and C−O−C in epoxide group. In the curve of PPD, the appearance of two peaks at about 3417 cm^−1^ were assigned to the stretching vibration of -N-H of NH_2_. The peaks at 1623 cm^-1^ and 1520 cm^−1^ were attributed to the bending vibration of N-H of NH_2_. Compared with the curves of PPD and GO, there were two new peaks at 1173 cm^−1^ and 727 cm^−1^ appeared on the curve of PPD-GO, which corresponding the stretching vibration of C−N and the stretching vibration of N-H of C-NH-, respectively [34,35]. Meanwhile, the peak at 3210 cm^−1^ was attributed to the H-bond interaction between −NH_2_ and oxygen-containing groups of GO [28]. It indicated the formation of C−NH−C bands due to the grafting of PPD to GO surface.

**Figure 3:**
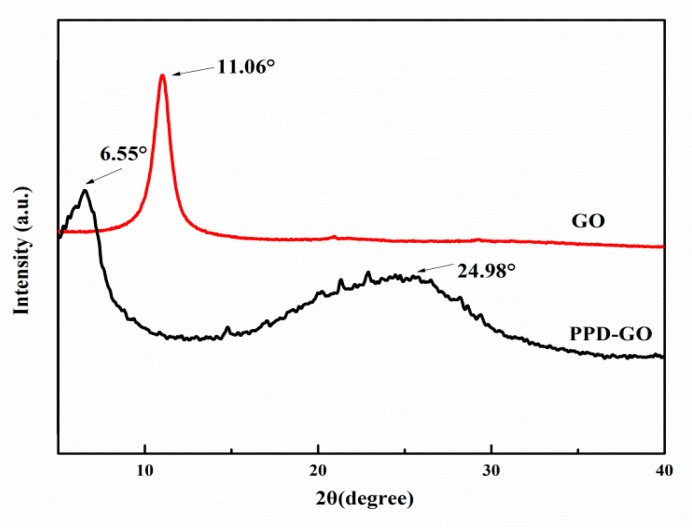
X-ray diffractograms of the GO and PPD-GO.

In X-ray diffractograms (Figure 3), there was only one peak on the spectra of GO at 11.06°, corresponding the crystal face (002). After PPD was grafted onto GO, the crystal face (002) transferred to 6.55° and the basal spacing (002) increased from 0.85 nm to 1.35 nm, indicating that a certain amount of PPD entered the interlayer of GO. It was noting that there was a broad peak on the spectra of PPD-GO at 24.98°, which coincided with the characteristic peak of graphene, indicating that PPD had a reduction effect on GO. In the curve of PPD-G, there were many small peaks between the 2*θ* of about 15°–30°, indicated that GO was exfoliated to some extent and PPD formed small polymeric crystallites on the GO sheets [36].

**Figure 4:**
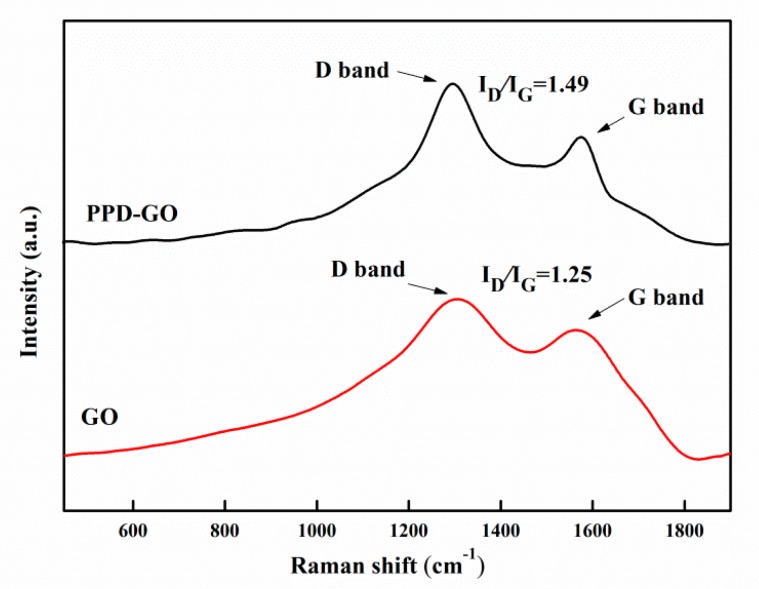
Raman spectra of GO and PPD-GO.

The intensity ratio (I_D_/I_G_) of the D-peak to G-peak of the Raman spectra of graphene is commonly used to characterize the defect density of graphene [37,38,39,40]. In the Raman spectra of GO and PPD-GO (Figure 4), D peak and G peak appeared around 1310 cm^−1^ and 1580 cm^−1^ respectively. The ID/IG of PPD-GO was 1.49, which was 0.24 higher than that of GO. This is because the amino group of PPD reacted with the oxygen-containing group on GO, resulting in the decrease of the average size of the sp^2^ domain [30].

### 3.2. The Mechanical Properties of SSBR Composites Before and After Aging.

**Figure 5:**
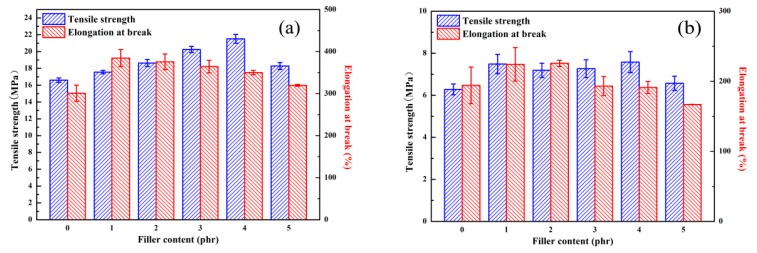
Tensile strength and elongation at break of solution polymerized styrene-butadiene rubber (SSBR) composites with different PPD-GO content before aging (**a**) and after aging (**b**).

Tensile tests were conducted to evaluate the mechanical properties of SSBR composites. It can be seen from Figure 5a and Table 3 that SSBR/PPD-GO composites had better mechanical properties than SSBR without PPD. In particular, the tensile strength and the elongation at break of SSBR/PPD-GO4 were 21.5 MPa and 350%, respectively. When the amount of PPD-GO reached 5 phr, the mechanical properties of SSBR/PPD-GO decreased to the values comparable to those for SSBR. This is due to the fact that PPD-GO agglomerated in the SSBR matrix when the amount of PPD-GO increased, resulting in a decrease in mechanical properties. In order to investigate the effect of PPD-GO on the thermo-oxidative aging resistance, SSBR composites were selected to conduct the thermal-oxygen aging test at 90 °C for 96 h. After thermal oxygen aging, mechanical properties of rubber will change significantly. In Figure 5b and Table 3, the elongation at break of SSBR/PPD-GO composites was almost equivalent to that of SSBR, which showed a slight trend of increasing first and decreasing later. The tensile strength of SSBR/PPD-GO composites was about 1.3 MPa higher. It was indicated that PPD-GO can not only significantly improve the mechanical properties of SSBR, but also effectively enhance the aging resistance of SSBR.

### 3.3. The Thermal Properties of SSBR Composites

**Figure 6:**
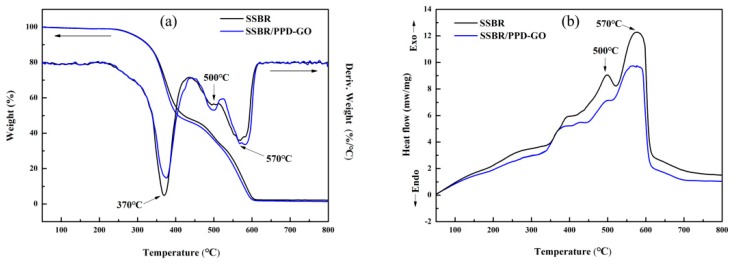
Thermogravimetric-differential scanning calorimeter synchronization analysis (TGA-DSC) curves of SSBR and SSBR/PPD-GO under air atmosphere. (**a**) The TGA curve; (**b**) The DSC curve.

The synchronous thermal analysis of SSBR composites was tested under oxygen atmosphere. The content of PPD-GO was 4 phr. In Figure 6a, the TGA curve of SSBR composites was mainly divided into two stages. The first stage was from 200 °C to 440 °C, which mainly attributed to the primary degradation of rubber chains. The second stage was from 440 °C to 600 °C, which attributed to the further degradation of rubber and thermal oxidation degradation of carbon black. In the first stage, the temperature corresponding to the maximum degradation rate of SSBR/PPD-GO was 10 °C higher than that of SSBR. This is because some of the free radicals generated in the thermal oxygen degradation process were captured by the amino group on PPD-GO [6]. Figure 6b showed the DSC curve of SSBR/PPD-GO. The exothermic peak of SSBR in the whole degradation process was more obvious than that of SSBR/PPD-GO, indicating that the PPD-GO effectively slowed down the thermal oxygen degradation of SSBR. 

### 3.4. The Micromorphology of SSBR Composites

**Figure 7:**
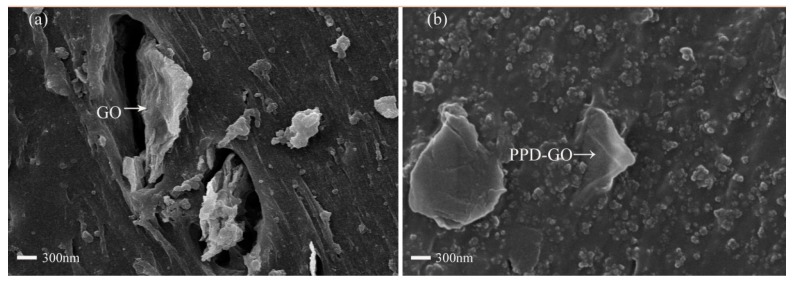
Scanning electron microscopy (SEM) images of fractured surfaces of SSBR/GO (**a**) and (**b**) SSBR/PPD-GO4.

Variations in the surface morphology of SSBR composites were illustrated by SEM. In Figure 7a, there were obvious pores between GO and the SSBR matrix. In Figure 7b, the PPD-GO was embedded in the fracture surface and maintained a good interaction with the SSBR matrix. It can be attributed to the grafting of PPD, which weakened the agglomeration of GO.

### 3.5. The Dynamic Mechanical Properties of SSBR Composites

**Figure 8:**
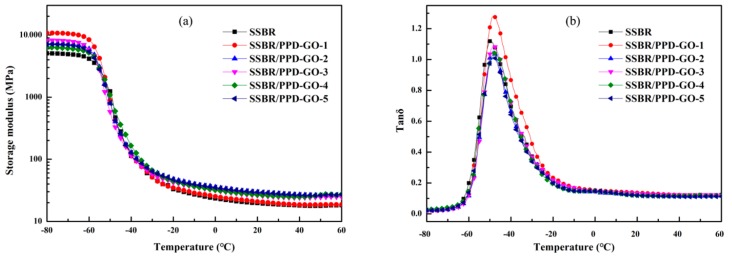
Storage modulus (**a**) and tanδ (**b**) curves of SSBR composites.

It can be seen from Figure 8 that the storage modulus (E’) of SSBR/PPD-GO composite was higher than that of the SSBR and the peak in mechanical loss factor (tan*δ*) of SSBR/PPD-GO composites moved to high temperature, indicating that the crosslinking density of SSBR/PPD-GO composite was more than that of SSBR. This was due to the strong interaction between PPD-GO and SSBR matrix, which formed many physical crosslink points [41,42,43]. 

The tanδ value at 0 °C and 60 °C was the indicator for evaluation of high performance rubber tire materials [44]. It can be considered that the larger the tanδ value at 0 °C, the better wet-skid resistance, and the smaller the tan*δ* value at 60 °C, the less the rolling loss. As shown in Table 4, the tan*δ* value of SSBR/PPD-GO composites at 0 °C was increased. The tan*δ* value of SSBR/PPD-GO1 was 0.153, an increase of 5.5% over that of SSBR. At 60 °C, the tan*δ* value of SSBR/PPD-GO composites was decreased with the content of PPD-GO. The tanδ value of SSBR/PPD-GO5 was the smallest, which was 8.9% lower than that of SSBR. This indicated that it had a good effect on improving the wet-skid resistance with the low content of PPD-GO and a positive contribution on reducing the rolling loss with the high content of PPD-GO. Both of wet-skid resistance and low rolling loss of SSBR/PPD-GO4 had been obviously improved, which were 2.1% and 6.5% higher than that of SSBR without PPD-GO.

## 4. Conclusions

In this study, GO grafted by PPD was added into SSBR to give SSBR/PPD-GO composites. The interaction between GO and the SSBR matrix was enhanced by introducing PPD. PPD-GO can be considered the good particle to increase the mechanical properties and aging resistance of SSBR. When the content of PPD-GO was 4 phr, the tensile strength and elongation at break were 21.5 MPa and 350% respectively, which were 29.5% and 16.7% higher than those of SSBR. After aging, the mechanical properties of SSBR/PPD-GO composites were also better than that of SSBR, in which the tensile strength was about 1.3 MPa higher. PPD-GO effectively improved the thermal oxidative aging resistance of SSBR. The better wet-skid resistance and lower rolling loss of SSBR was increased by adjusting the content of PPD-GO. In particular, both of wet-skid resistance and low rolling loss of SSBR/PPD-GO4 had been improved, which were 2.1% and 6.5% higher than that of SSBR respectively. This research can be thought of as an effective method to obtain the SSBR composite with good integrated properties in aging resistance, wet-skid resistance and low rolling loss.

## Figures and Tables

**Table 1:** The list of experimental formula (parts per hundreds of rubber).

Sample	SSBR	ZnO	Stearic Acid	Sulfur	TBBS	Carbon Black	PPD-GO
SSBR	100	2.18	0.73	1.27	1.00	50.00	0
PPD-GO1	100	2.18	0.73	1.27	1.00	50.00	1.00
PPD-GO2	100	2.18	0.73	1.27	1.00	50.00	2.00
PPD-GO3	100	2.18	0.73	1.27	1.00	50.00	3.00
PPD-GO4	100	2.18	0.73	1.27	1.00	50.00	4.00
PPD-GO5	100	2.18	0.73	1.27	1.00	50.00	5.00

**Table 2:** The FTIR bands assignments.

GO		PPD		PPD-GO	
Wavenumber(cm^−1^)	Group	Wavenumber(cm^−1^)	Group	Wavenumber(cm^−1^)	Group
3423	-OH	3417	-NH	3210	H-bond
1721	C=O	1623, 1520	N-H of NH_2_	1173	C−N
1620	C=C	-	-	727	N-H of C-NH-
1404	-COOH	-	-	-	-
1048	C-O-C	-	-	-	-

**Table 3:** Mechanical properties SSBR composites before and after aging.

Sample		SSBR	PPD-GO1	PPD-GO2	PPD-GO3	PPD-GO4	PPD-GO5
tensile strength (Mpa) elongation at break (%)	before aging	16.6 ± 0.3	17.6 ± 0.2	18.6 ± 0.4	20.3 ± 0.4	21.5 ± 0.5	18.3 ± 0.4
	after aging	6.3 ± 0.3	7.5 ± 0.5	7.9 ± 0.3	7.3 ± 0.4	7.6 ± 0.5	6.6 ± 0.3
	before aging	301 ± 19	384 ± 20	375 ± 18	364 ± 14	350 ± 5	319 ± 2
	after aging	194 ± 26	224 ± 23	225 ± 4	193 ± 13	181 ± 8	166 ± 2

**Table 4:** The tanδ of SSBR/PPD-GO composites.

Sample	SSBR	PPD-GO1	PPD-GO2	PPD-GO3	PPD-GO4	PPD-GO5
0 °C	0.145	0.153	0.145	0.146	0.148	0.147
	±0.0002	±0.0001	±0.0002	±0.0001	±0.0001	±0.0002
60 °C	0.123	0.119	0.116	0.120	0.115	0.112
	±0.0001	±0.00023	±0.0002	±0.0003	±0.0001	±0.0001
